# Simulation Training and Peripheral Angioplasty Learning: Protocol of a Quasi-Experimental Evaluative Cross-Sectional Study

**DOI:** 10.2196/64658

**Published:** 2026-07-27

**Authors:** Rania Hammami, Emna Derbel, Wafa Elleuch, Rahma Kallel, Aimen Dammak, Faiza Safi, Fatma Mhiri, Hichem Denguir, Mootez Billah Oueslati, Souad Ferjani Milouchi, Sami Milouchi, Faten Dhouib, Naziha Turki

**Affiliations:** 1 Cardiology Department, Simulation Center, Faculty of Medicine of Sfax, Military University Hospital of Sfax, Military University Hospital of Sfax, Sfax, MM66+874, Tunisia, 216 24056985; 2 Pedagogy Department, Faculty of Medicine of Sfax, Sfax, Tunisia; 3 Cardiology Department, Simulation Center, Faculty of Medicine of Sfax, Gabes Hospital, Gabes, Tunisia; 4 Cardiovascular Surgery, Simulation Center, Faculty of Medicine of Sfax, Habib Bourguiba Hospital, Sfax, Tunisia; 5 Pediatric Department, Simulation Center, Faculty of Medicine of Sfax, Hedi Chaker Hospital, Sfax, Tunisia; 6 Ministry of Health, Tunis, Tunisia; 7 Radiology Department, Simulation Center, Faculty of Medicine of Sfax, Habib Bourguiba Hospital, Medenine, Tunisia; 8 Cardiology Department, Simulation Center, Faculty of Medicine of Sfax, Habib Bourguiba Hospital, Medenine, Tunisia; 9 Cardiovascular Surgery Department, Faculty of Medicine of Sfax, Military University Hospital of Sfax, Sfax, Tunisia

**Keywords:** radioprotection, simulation, scores, learning, peripheral angioplasty

## Abstract

**Background:**

Simulation-based training has demonstrated effectiveness in enhancing proficiency in interventional procedures. The endovascular treatment of peripheral arterial lesions has become a prominent minimally invasive therapeutic option. However, operators from various specialties, including Interventional Cardiology, Interventional Radiology, and Vascular Surgery, often follow diverging guideline recommendations and training pathways. Despite growing use of simulation in this field, the influence of an operator’s specialty on learning outcomes remains poorly understood.

**Objective:**

This study aimed to evaluate the impact of simulation training on peripheral angioplasty performance scores across different interventional specialties and assess how specialty-specific factors influence skill acquisition.

**Methods:**

This quasi-experimental observational study will be conducted at the Simulation Center of the Faculty of Medicine of Sfax, Tunisia (trial registration: PACTR Trial ID 30697). Participants consist of 60 novice fellows from three specialties: Cardiology (n=20), Interventional Radiology (n=20), and Vascular Surgery (n=20). All participants will undergo identical simulation training using a high-fidelity Mentice simulator, comprising five standardized clinical scenarios addressing peripheral angioplasty of complex iliac and femoral lesions (TASC B-D). Each participant will receive a 4-hour hands-on training session with pre- and posttest assessments using a 25-item Global Score (10 knowledge items, 15 competence items). Primary outcomes consist of percentage change in performance scores from baseline to posttraining (threshold for clinical significance:≥25% improvement) and comparison across specialty groups using nonparametric statistics. Secondary outcomes consist of radiation protection parameters, including fluoroscopy duration, cumulative radiation dose, air kerma, and dose area product (KAP), measured by a simulator with real-time ALARA (As Low As Reasonably Achievable) feedback.

**Results:**

Study enrollment started on September 15, 2024. As of December 2025, 42 fellows were screened, and 21 have been enrolled (15 Cardiology, 3 Radiology, 3 Vascular Surgery), representing 35% of the target sample (21/60). All 21 enrolled participants completed the full simulation training protocol, with radiation protection parameters recorded for all sessions. Enrollment and data collection are expected to be completed by June 2026, with full results anticipated for publication thereafter.

**Conclusions:**

This protocol describes the first comparative study evaluating the impact of high-fidelity simulation on peripheral angioplasty performance across three distinct specialties. Findings are anticipated to provide novel evidence regarding specialty-specific learning trajectories and may inform the development of standardized, specialty-tailored training pathways for complex peripheral interventions.

## Introduction

Peripheral arterial disease (PAD) represents a significant and growing health burden, with recent epidemiological data demonstrating a 72% increase in global prevalence over the past decade [1,2]. The endovascular treatment of peripheral arterial lesions has emerged as the preferred minimally invasive therapeutic approach for many patients, particularly those at high surgical risk [2]. Peripheral angioplasty for complex iliac and femoral lesions—particularly those classified as TASC (TransAtlantic InterSociety Consensus) II Grade C and D lesions—demands advanced technical skills, precise anatomic knowledge, and procedural expertise. Simulation-based training has shown considerable promise in enhancing procedural competence and clinical outcomes in interventional procedures [3-7]. Multiple studies have demonstrated that high-fidelity simulation training can reduce complications, improve patient safety, decrease procedural time, and minimize radiation exposure in real-world clinical practice [5-7]. Beyond these clinical benefits, simulation provides a low-risk environment for deliberate practice and skill acquisition in complex procedures. However, a critical gap exists in our understanding of how a specialty background influences learning outcomes in simulation-based interventional training. Fellows from Cardiology, Interventional Radiology, and Vascular Surgery often approach peripheral angioplasty through different clinical lenses, guided by their respective specialty societies’ divergent guidelines and training pathways. Although these three specialties frequently encounter PAD and perform endovascular interventions, they typically follow different procedural approaches, use distinct terminology, and emphasize different clinical endpoints [8]. Prior research by Wallace et al and Soenens et al suggested that outcomes of endovascular interventions may depend more on clinical indication than on physician specialty [9,10]. However, this finding does not address whether a specialty background influences the acquisition of technical skills through simulation training. Furthermore, while several studies have documented that vascular surgery residents improve endovascular procedure skills through high-fidelity simulation training [3,6], corresponding evidence for Cardiology and Radiology trainees remains limited.

We hypothesize that despite receiving identical simulation-based training content and learning objectives, fellows from different specialty backgrounds will demonstrate differential learning trajectories and performance outcomes. Specifically, we postulate that all three specialty groups will demonstrate significant performance improvement following simulation training; however, Cardiology fellows may demonstrate slightly superior performance scores due to greater baseline exposure to endovascular techniques through coronary catheterization experience [7]. This hypothesis is grounded in educational literature demonstrating that learners with different prior experiences assimilate novel information through different cognitive schemas and may benefit from specialty-tailored educational approaches. Understanding these specialty-specific differences could guide the development of more effective, targeted simulation curricula across specialties.

The main aim of this study is to evaluate the impact of high-fidelity simulation training on peripheral angioplasty performance skills and learning outcomes across three distinct interventional specialties (ie, Cardiology, Interventional Radiology, and Vascular Surgery).

## Methods

### Study Design

This is a quasi-experimental observational, cross-sectional evaluative study that is conducted at the Simulation Center of the Faculty of Medicine of Sfax from September 15, 2024, to June 2026. We are assessing the effectiveness of a training session using an interventional simulator in the learning of invasive procedures (ie, peripheral angioplasty) among beginners in the field.

This is a quasi-experimental, observational, cross-sectional evaluative study. The study was registered with PACTR (TrialID=30697) and received ethical approval from the Ethics Committee of the Faculty of Medicine of Sfax (approval number 49/24).

### Study Setting and Recruitment

- Physical setting: The study is being conducted at the Simulation Center of the Faculty of Medicine of Sfax, Tunisia, a dedicated educational facility equipped with high-fidelity interventional simulation equipment and training infrastructure. The included fellows were either participants in the Interventional Cardiology Diploma program organized by the Faculty of Medicine of Sfax, or fellows currently undertaking clinical rotations in the Department of Cardiology at the Military Hospital of Sfax, the Department of Cardiac Surgery at Habib Bourguiba University Hospital, the Department of Cardiology in Gabès, the Department of Cardiology in Médenine, or the Department of Radiology in Médenine.
- Participant eligibility and screening*:* The participants are fellows who have never performed or participated in an interventional procedure of peripheral vascular disease, and were divided into 3 groups: fellows in Cardiology (n=20), fellows in Radiology (n=20), and fellows in Vascular Surgery (n=20).
  Inclusion criteria comprised active fellows enrolled in accredited Cardiology, Interventional radiology, or Vascular surgery fellowship programs who had no prior experience performing or assisting in peripheral vascular interventional procedures (novice operators). Participants were required to provide written informed consent and to be available to complete the entire 5-day simulation-based training program, including mandatory pre- and posttraining assessments.
  Noninclusion criteria included refusal to provide written informed consent, any prior exposure to peripheral interventions in the catheterization laboratory, or previous participation in simulation-based training for peripheral vascular disease.
  Exclusion criteria were defined as failure to complete the full simulation training protocol or the presence of incomplete assessment data, including missing pre-test or post-test evaluations.
- Screening and enrollment process*:* All eligible fellows who express interest complete a screening questionnaire assessing prior experience. Screened participants who meet the inclusion criteria are provided with detailed information about the study and training protocol. Those willing to participate provide written informed consent. All participants are novice operators regarding peripheral angioplasty but may have prior exposure to other interventional procedures (eg, coronary angiography for Cardiology fellows).

### Simulation Training Curriculum and Procedure

In this study, all three groups of learners—fellows in Cardiology, Radiology, and Vascular Surgery—are undergoing the same standardized simulation training curriculum.

- Training protocol overview*:* The training program was conducted over 3 steps following a standardized and reproducible structure.
  Step 1 was dedicated to orientation and demonstration. Participants first completed orientation questionnaires to assess baseline experience and familiarity with endovascular procedures. An experienced operator (physician with more than five years of simulation expertise) then introduced the simulator components and functionalities, followed by a live demonstration of complete diagnostic angiography and peripheral angioplasty procedures. At the end of this step, each participant performed an initial pretest assessment, establishing a baseline Global Score. Step 2 consists of a 4-hour individual training session. During this session, all participants practiced the same set of five standardized clinical scenarios, ensuring uniform exposure and learning conditions. Continuous, real-time feedback was provided by a single dedicated trainer to maintain pedagogical consistency, with a trainer-to-participant ratio of 1:1 throughout all training sessions. In step 3, after completion of the training program, participants underwent a posttraining assessment, using the same scenarios and evaluation metrics as the pretest, to generate a posttest Global Score. All participants completed five standardized clinical training scenarios, each designed with identical educational objectives and arranged in a progressive sequence of increasing complexity, ranging from simple to more advanced peripheral vascular pathology, as detailed in Table 1. All scenarios are conducted in identical hemodynamic conditions and with standardized anatomical variations. The order is fixed across all participants to ensure consistency. All learners were supervised by the same trainer.

The study used a “Mentice Brand Simulator,” an interventional simulator with a high-fidelity platform. The system incorporates an advanced hardware configuration, including a control unit enabling adjustment of fluoroscopic projection angles, table movements, and image magnification; a fluoroscopy pedal allowing real-time fluoroscopic activation; an instructor module permitting selection among a wide range of catheters, guidewires, balloons, and stents; and a haptic feedback system that provides realistic tactile sensations during device manipulation. The simulator is supported by an integrated software and display system based on a notebook computer interface that presents detailed procedural and scenario information, real-time fluoroscopy duration, simulated patient hemodynamic parameters (including blood pressure and heart rate), cumulative radiation dose calculations, and anatomically accurate vascular models incorporating realistic lesion morphology and pathological features. In addition, the Mentice VIST-FLEX includes a comprehensive radiation simulation and safety monitoring module, offering realistic dose calculations such as fluoroscopy time and cumulative radiation dose.

**Table 1.** Learning objectives of selected cases (from the Mentice simulator manual).

Case	Type	Description	Sample training objective
202	Iliac	Right mid internal iliac	Lesion location and treatment site selection
203	Iliac	Left mid common iliac	Selective angiography of contralateral leg
204	Iliac	Right proximal external	Optimizing torque anatomy
206	Iliac	Left proximal external	Treatment options/risks
207	Iliac	Left external common iliac	Negotiating tortuous aorta

- Trainer Standardization*:* To eliminate instructor variability, all participants are trained by a single experienced interventional cardiologist (>10 years clinical experience,>5 years simulation training experience) using standardized feedback scripts and assessment protocols. The trainer follows a standardized feedback protocol consisting of: real-time procedural guidance, error correction with explanation of the correct approach, positive reinforcement for correct techniques, and structured feedback at the end of each scenario.
- Learner Evaluation and Scoring System*:* Learners were assessed at two time points by calculating a Global Score. This score comprised 25 items: 10 items for evaluating the knowledge of the participants (Knowledge score), 15 items for evaluating the skills (Competence score). These scores were calculated at the beginning of the training before the first procedure. The day after the training, the learner returned for the posttest, involving the repetition of the same procedures with which they started (Multimedia Appendix 1).

### Primary Outcome

Primary outcome is the percentage change in Global Score from pre-test to posttest, calculated as percentage improvement=100×(Final Global Score − Initial Global Score) / Initial Global Score. We will consider that there is a significant improvement in the knowledge and the skills of the learner if the percentage variation is more than 25%. We will also compare scores based on the specialty of the trainee: Cardiology, Radiology, and Vascular Surgery.

### Secondary Outcomes

As secondary outcomes, we will assess the radioprotection parameters. All radiation protection parameters are automatically recorded by the Mentice simulator for each participant’s each scenario:

1. Fluoroscopy duration (seconds): Total cumulative fluoroscopy time per procedure
2. Cumulative radiation dose (mGycm²): Total dose area product (KAP) accumulated during procedure
3. Air kerma (mGy): Absorbed radiation dose
4. Kerma area product (KAP) (Gycm²): Integrated dose across field area
5. Procedure duration (minutes): Total time from initial guidewire placement to final angiography
6. ALARA (As Low As Reasonably Achievable) compliance: Composite measure assessing adherence to radiation safety principles based on fluoroscopy efficiency (total fluoroscopy time/procedure duration ratio)

### Statistical Analysis

We will use SPSS Statistics software (version 21; IBM Corp) to conduct the statistical analysis. We will check the normality of the distribution of variables. Due to the small sample size, we expect that the distribution of our population is non-Gaussian. In this case, we will express all quantitative values as median (with extremes and the semiinterquartile range), and use nonparametric tests, specifically the Wilcoxon test, to compare performance and competence scores before and after the training cycle (paired sample tests). To compare scores between those who have never performed interventional procedures (novices) and those with prior experience, we will use the nonparametric Mann-Whitney *U* test. We will consider differences to be significant if *P*<.05.

### Ethical Considerations

#### Human Subject Ethics Review Approvals

This study was reviewed and approved by the Ethics Committee of the Faculty of Medicine of Sfax (IRB approval number: 49/24, approval date: July, 10, 2024). The protocol adheres to the Declaration of Helsinki and Good Clinical Practice guidelines. No amendments to the protocol have been implemented without prior ethics approval.

#### Informed Consent

All participants provide written informed consent prior to enrollment. The informed consent form clearly describes: study objectives and procedures, time commitment required, potential risks (minimal risk; study involves simulation training only, no actual patient care), confidentiality protections and the right to withdraw from study at any time without penalty. Participants will receive a signed copy of the informed consent form for their records.

#### Privacy and Confidentiality

All participant data is deidentified using unique study ID numbers. Data will not be shared with individuals outside the research team, and upon publication, no individual-level data will be presented that could identify participants.

#### Participant Compensation

Participants received no direct monetary compensation. However, compensation includes free high-quality simulation training and a certificate of completion upon successful training. All recorded materials will be stored on secure, encrypted servers accessible only to the research team; no identifying information of individual participants will appear in any manuscript or presentation materials.

## Results

### Study Enrollment and Participant Flow

Study enrollment commenced on September 15, 2024, at the Simulation Center of the Faculty of Medicine of Sfax, Tunisia. The study is planned over 21 months, with completion anticipated in June 2026.

As of December 2025, 42 fellows have been screened. Of these, 21 met the inclusion criteria and were enrolled, while 21 were excluded due to prior catheterization laboratory experience. The current enrollment corresponds to 35% (21/60) of the planned sample size.

### Training Completion Status

All 21 enrolled participants have completed the full five-scenario simulation training protocol, including both pretest and posttest assessments.

### Enrolled Participant Characteristics

Table 2 summarizes the characteristics of enrolled participants by specialty.

**Table 2.** Enrolled participant characteristics.

Specialty	Participants (n)	Sex (M:F) ratio	Age, mean (SD)	Year of Fellowship
Cardiology	15	13:2	28.4 (2.1)	PGY^a^-3 to PGY-5
Radiology	3	2:1	28.9 (1.8)	PGY-2 to PGY-5
Vascular Surgery	3	3:0	29.7 (1.5)	PGY-2 to PGY-5
Total	21	18:3	28.6 (2.3)	—

a PGY: postgraduate year.

### Study Timeline and Future Milestones

Completed milestones include the pilot study (HE Marman, MD, unpublished data, 2024), ethics approval from the Faculty of Medicine of Sfax (approval number 49/24), trainer standardization (August 2024), opening of enrollment (September 15, 2024), and enrollment and training of the first 21 participants.

Ongoing activities as of December 2025 consist of continued recruitment to reach the target sample size of 60 fellows, with focused efforts to increase participation specifically from Interventional Radiology and Vascular Surgery programs.

Planned milestones include completion of enrollment and data collection by June 2026, followed by statistical analysis during June-July 2026, and manuscript preparation and submission to JMIR Research Protocols during July-September 2026.

## Discussion

### Anticipated Principal Findings

Based on our methodological approach, this study is anticipated to demonstrate that high-fidelity simulation training significantly improves both knowledge and technical competence in peripheral angioplasty across all three specialty groups. The comparative analysis across specialties will be novel in the simulation training literature and will provide the first evidence specifically addressing whether specialty background influences skill acquisition in peripheral angioplasty simulation training.

### Comparison to Prior Work

#### Previous Research on Simulation Training

Prior studies have demonstrated the effectiveness of high-fidelity simulation in improving outcomes for various interventional procedures. Kreiser et al [11] recently reviewed the evidence for simulation training in Interventional radiology, highlighting improved procedural competence and reduced procedure times. Campbell et al [4] specifically addressed training challenges in PAD management. Dawson et al [12] provided early evidence that training with high-fidelity endovascular procedure simulators improves vascular surgery residents’ endovascular procedure skills, with documented improvements in procedure time and complication rates.

#### Specialty-Specific Variations

This study builds on these foundations by explicitly examining specialty-specific variations, which have not been previously systematically studied in simulation-based peripheral angioplasty training. The existence of divergent society guidelines for peripheral artery disease management has been documented by Tan et al [13], who compared recommendations from the Society for Vascular Surgery, American Heart Association/American College of Cardiology, and European Society for Vascular Surgery. These divergences in clinical approach may manifest as different learning trajectories when specialists undergo identical simulation-based training.

Wallace et al [9] previously suggested that outcomes of endovascular interventions depend more on clinical indication than on physician specialty. This study addresses a complementary question: whether physician specialty influences the acquisition of technical skills through simulation training, independent of actual clinical outcomes.

### The Evolution of Vascular Interventional Practice

Szőnyi et al [14] have recently reviewed the evolution of vascular interventional radiology and endovascular surgery, noting the increasing involvement of multiple specialties in peripheral interventions and the resulting need for standardized training pathways. This study directly addresses the identified need.

### Study Strengths

This study demonstrates several key strengths, including the use of high-fidelity simulation with comprehensive radiation dosimetry monitoring—unlike previous studies focused primarily on procedural competence—which aligns with current ALARA principles; a standardized curriculum delivered identically across all three specialty groups that eliminates content confounding and isolates specialty-related learning factors; employment of a single experienced trainer throughout to ensure consistent assessment criteria and eliminate instructor variability; a prospective pre-test or post-test design comparing each participant to their own baseline for robust evidence of training effectiveness; a novel multi-specialty comparative design representing the first systematic evaluation of learning outcomes across Cardiology, Radiology, and Vascular surgery fellows in peripheral angioplasty simulation training; comprehensive 25-item Global Score assessment instruments evaluating both knowledge (10 items) and technical competence (15 items); and detailed methodology documentation enabling replication by other simulation centers.

### Study Limitations

The primary limitations include absence of real-world validation through direct assessment of participant performance in actual catheterization laboratory settings, which limits clinical applicability; current enrollment of 21/60 participants with unequal specialty distribution (particularly only 3 radiology fellows) that constrains statistical power for between-group comparisons; and the single-center design conducted solely at the Faculty of Medicine of Sfax, which may limit generalizability to other geographic regions, health care systems, or simulation centers with different equipment and institutional cultures.

### Conclusion

This protocol describes the first comparative study evaluating the impact of high-fidelity simulation training on peripheral angioplasty skill acquisition and performance outcomes across three distinct interventional specialties: cardiology, interventional radiology, and vascular surgery. The study will also provide comprehensive data on radiation protection parameters, contributing to the evidence base for ALARA principle implementation in procedural training. Upon completion, this study will serve as a methodological template for examining specialty-specific learning across diverse interventional procedures and specialties, supporting evidence-based approaches to procedural training and competency assessment in the future.

## Supplementary material

10.2196/64658 Multimedia Appendix 1 Global Score Structure (0-100 points).

## Abbreviations

ALARA: As Low As Reasonably Achievable
IRB: institutional review board
KAP: Kerma Area Product
mGy: Milligray
mGycm²: Milligray-centimeters squared
PAD: peripheral arterial disease
PGY: postgraduate year
TASC: TransAtlantic InterSociety Consensus
