# Supplementary material for: Simulation Training and Peripheral Angioplasty Learning: Protocol of a Quasi-Experimental Evaluative Cross-Sectional Study
Source: JMIR Res Protoc. 2026 Jul 27;15:e64658. doi: 10.2196/64658 (PMC13404036; doi:10.2196/64658)
Supplement: Supplementary file 1 [file resprot-v15-e64658-s001.odt]

##  Global Score Structure (0-100 points): The evaluation instrument consists of 25 items divided into two components:

| Knowledge Score:  assesses conceptual understanding of peripheral angioplasty principles:(10 items; 40% of global score; maximum 40 points) |
| --- |
| Peripheral artery disease classification (0-10 points): | Understanding of Fontaine classification, TASC grading system, and the anatomic distribution of PAD |
| TASC staging and complexity assessment (0-10 points): | Ability to correctly identify and classify lesion complexity from imaging and clinical presentation |
| Indication and contraindication knowledge (0-10 points): | Understanding of appropriate indications for endovascular vs. surgical treatment, and procedural contraindications |
| Anatomy and access considerations (0-10 points): | Knowledge of vascular anatomy relevant to peripheral intervention, including common access routes and anatomic variants |
| Equipment principles (0-10 points): | Understanding of catheter types, guidewire properties, balloon characteristics, and stent selection principles |
| Radiation safety and ALARA principles (0-10 points): | Knowledge of radiation physics, dose-response relationships, and methods to minimize exposure |
| Hemodynamic monitoring (0-10 points): | Understanding of vital sign responses to procedures and medications |
| Complication recognition (0-10 points): | Knowledge of common complications and early recognition signs |
| Pharmacology basics (0-10 points): | Knowledge of anticoagulation, antiplatelet therapy, and vasodilators used |
| Clinical outcomes (0-10 points): | Understanding of expected outcomes, success definitions, and long-term patency data |
| Competence Score: assesses technical and procedural skills(15 items; 60% of global score; maximum 60 points) |
| Vascular Access Techniques (2 items) |  |
| Choice of vascular artery access (0-10 points) |  |
| Guidewire positioning and manipulation (0-10 points): | Gentle wire handling, appropriate positioning in target vessel, management of wire tortuosity |
| Diagnostic Angiography (3 items) |  |
| Projection/incidence selection (0-10 points): | Appropriate fluoroscopy angles to visualize lesion without foreshortening |
| Field of view optimization (0-10 points): | Proper magnification and field positioning to visualize relevant anatomy without unnecessary radiation |
| . Lesion characterization (0-10 points): | Systematic lesion assessment identifying occlusion vs. stenosis, length, calcification, and involvement of branch vessels |
| Interventional Techniques (7 items) |  |
| 6. Lesion crossing strategy (0-10 points): | Appropriate selection of crossing technique (subintimal vs. intraluminal) based on lesion characteristics |
| 7. Wire and catheter positioning (0-10 points): | Precise positioning in target vessel avoiding side branch injury or perforation |
| 8. Balloon sizing and positioning (0-10 points): | Appropriate balloon diameter selection and correct positioning across the lesion |
| 9. Balloon inflation technique (0-10 points): | Appropriate pressure and duration for effective angioplasty |
| 10. Stent deployment (0-10 points, when applicable): | Correct stent size selection, positioning, and deployment technique |
| 11. Post-intervention angiography (0-10 points): | Systematic assessment of post-procedure result, distal runoff, and evidence of complications |
| 12. Vascular closure (0-10 points): | Appropriate hemostasis technique and wound management |
| Procedural Safety (3 items) |
| 13. Fluoroscopy time optimization (0-10 points): | Minimal fluoroscopy use, appropriate use of last-image-hold function, rapid procedure execution |
| 14. Radiation dose minimization (0-10 points): | Strict adherence to ALARA principles, minimum magnification necessary, minimal fluoroscopy runs |
| 15. Procedure time efficiency (0-10 points): | Organized approach, minimal repositioning, efficient use of available time |
| Scoring Rubric: Each item scored on a 10-point Likert scale: |
| 9-10 points: | Excellent/Expert performance (meets all criteria, smooth execution, independent performance) |
| 7-8 points: | Good performance (meets most criteria, minor deviations, requires minimal guidance) |
| 5-6 points: | Acceptable/Satisfactory performance (meets basic criteria, multiple minor deviations or occasional major deviations, requires some guidance) |
| 3-4 points: | Below acceptable/needs improvement (significant deviations from criteria, requires considerable guidance) |
| 0-2 points: | Inadequate/unsatisfactory performance (fails to meet criteria, incomplete understanding) |
| The Global Score was calculated as the sum of the Knowledge Score and the Competence Score, with a maximum achievable total of 100 points, expressed as a percentage ranging from 0 to 100%. Score interpretation was predefined as follows: a Global Score of ≥80% was considered excellent, 60–79% good, 40–59% acceptable, and <40% inadequate. |
